# Nutrition, Digestive System, and Clinical Gastroenterology

**DOI:** 10.3390/nu18050733

**Published:** 2026-02-25

**Authors:** Jorge Fonseca

**Affiliations:** 1 Aging Lab, Egas Moniz Center for Interdisciplinary Research (CiiEM), Egas Moniz School of Health and Science, 2829-511 Almada, Portugal; jorgedafonseca@gmail.com; 2 GENE-Artificial NutritionTeam, Gastroenterology Department, Hospital Garcia de Orta, 2805-267 Almada, Portugal

This Special Issue in *Nutrients*, “Dietary and Nutritional Therapies to Improve Digestive Disorders,” focuses on the fundamental and increasingly recognized relationship between nutrition and digestive diseases. For clinicians managing gastrointestinal conditions—particularly gastroenterologists and digestive surgeons—clinical nutrition has become an integral component of guidelines and practice [1]. Gastroenterologists’ special commitment to addressing nutrition issues and further enhancing their skills was recognized long ago [2,3]. Recent scientific advances have strengthened the understanding of the bidirectional link between the digestive system and human nutrition, encompassing nutritional physiology and pathophysiology, the clinical course of digestive diseases, and endoscopic or surgical therapeutic strategies.

From a physiological standpoint, human nutrition is regulated through coordinated interactions between the digestive and central nervous systems [4,5,6]. The digestive tract ensures digestion and absorption, while the liver serves as the primary reservoir and regulator of energy–protein stores. As the central organ of metabolic homeostasis, the liver governs lipid, carbohydrate, and amino acid metabolism; synthesizes most serum proteins, capable of transporting nutrients [7]; produces cholesterol and other lipids [8]; and maintains glucose and energy availability through glycogenolysis, gluconeogenesis, and ketogenesis, core metabolic pathways for periods of fasting or very reduced food intake [9]. These processes are modulated by a complex network of hormones secreted by the gastrointestinal tract and accessory organs [10]. Advances in digestive endocrinology have now clarified how these hormonal pathways regulate appetite, satiety, and systemic metabolism in concert with hypothalamic centers [11,12,13,14,15].

In patients with acute and chronic digestive diseases, inflammatory and stress-mediated alterations in digestive hormone secretion contribute to anorexia and metabolic dysregulation [16]. Interactions between the intestinal immune system and the gut microbiota form a dynamic equilibrium whose disruption has been associated with low-grade inflammation [17,18], metabolic disorders [19], obesity [20,21], and even neurodegenerative diseases such as Parkinson’s disease, which initiates with the formation of alpha-synuclein aggregates in the intestinal wall, with secondary migration to the central nervous system, where they produce the typical neurological symptoms [22,23]. The digestive system comprising the gastrointestinal tract, microbiota, liver, pancreas, and mesenteric fat (called visceral fat but located around the digestive tract) [24] establishes a fruitful partnership with the central nervous system ruling human nutritional metabolism and thus functions as the central organ of human nutrition. Its dysfunction plays a pivotal role in the development of systemic disease. This Special Issue presents a very interesting assessment of these relationships, describing the gut–brain axis (GBA) as a bidirectional communication network between the gastrointestinal tract and the brain. This collection highlights the risk of malnutrition causing dysbiosis and GBA dysfunction and reminds us that various nutraceutical interventions can help restore balance, including probiotics, prebiotics, symbiotics, postbiotics, and paraprobiotics, which can modulate the gut microbiota and restore GBA homeostasis and metabolic and cognitive health [25].

Clinically, gastroenterologists and digestive surgeons routinely manage conditions that profoundly affect nutritional status [26,27]. Diseases that induce anorexia, prolonged fasting, restricted intake, malabsorption, or chronic inflammation frequently lead to malnutrition. Inflammatory bowel diseases (IBDs), particularly Crohn’s disease, exemplify this challenge, as reduced intake, impaired absorption, and inflammatory catabolism place patients at high nutritional risk [28]. Therefore, nutritional therapy, including exclusive diets and oral nutritional supplements, is essential [29,30]. This Special Issue includes two articles on the nutritional treatment of inflammatory bowel diseases, a clear demonstration of the importance of this intervention. One of these articles focuses on the nutritional management of children with Crohn’s disease, providing an extensive literature review of exclusive enteral nutrition, achieving a very high remission rate, with or without transforming growth factor-β (TGF-β) [31]. The other one provides a broader vision of IBD and irritable bowel syndrome (IBS) therapy, including not only diet, but also physical activity, cognitive behavioral therapies, and medical nutrition with probiotics, soluble fibers, and micronutrients [32]. Eosinophilic esophagitis is another disease for which treatment is often based on dietary intervention [33], as is the case for other hypersensitivity syndromes and allergic intestinal disorders. Malabsorption syndromes—whether due to celiac disease, eosinophilic gastrointestinal disorders, or other etiologies—systematically require targeted nutritional management [34].

Intestinal failure (IF) is defined as any clinical situation with reduced gut function below the minimum necessary for absorption of macronutrients and/or water and electrolytes. Intravenous supplementation is necessary for maintaining health, growth, body homeostasis, and survival. Several IF classifications are routinely used, but a basic one is the Functional Classification that defines three types, according to clinical presentation and evolution: Type I is acute, short-term, and often self-limiting, requiring intravenous support for a few days, for patients after abdominal surgery; Type II is prolonged failure, often in metabolically unstable patients with enteric fistulas, requiring multidisciplinary care and intravenous supplementation during weeks or months; Type III is chronic, in stable patients, requiring intravenous supplementation over months or years. In fact, Type III IF is the rarest form of chronic organ failure, and multimodal treatment of this disorder is complex [35,36]. Long-term home parenteral nutrition remains indispensable for patients with Type III IF, representing one of the most challenging chronic conditions in clinical gastroenterology [37,38]. This Special Issue of *Nutrients* presents an interesting pioneer experience of a country where the management of these patients is still rising [39]. Appetite and hyperphagia are associated with better outcomes in short bowel syndrome, the most common cause of Type III IF. This Special Issue includes an interesting article on the importance of oral diet in these patients [40], a major subject in medical management that cannot be overlooked.

Liver and pancreas disorders further underscore the tight link between digestive function and nutrition. Patients with chronic liver disease or cirrhosis face substantial nutritional risk [41], and malnutrition is a recognized predictor of adverse outcomes [42,43]. Chronic pancreatic disease poses dual challenges: maldigestion due to exocrine insufficiency and metabolic instability due to impaired endocrine secretion [44]. Conversely, nutritional disorders, particularly obesity, can contribute to pancreatic diseases, and this Special Issue also focuses on this subject, presenting a comprehensive review on the importance of obesity and metabolic dysfunction on the pathogenesis of acute and chronic pancreatitis and pancreatic cancer [45]. The relationship between nutrition and digestive oncology is equally significant. Dietary patterns and food contaminants influence carcinogenesis throughout the digestive system, either directly [46] or indirectly, through the modulation of gut microbiota [47]. Conversely, digestive cancers are among the malignancies most strongly associated with malnutrition.

Endoscopic interventions play a central role in addressing nutrition-related complications. Gastroenterologists routinely manage obstructive or motility-related feeding problems through stent placement, enteral tube insertion, and creation of gastrostomies or jejunostomies [48,49]. Selecting the most appropriate nutritional access route requires careful consideration of anatomical, functional, and prognostic factors [50]. Stent placement may be adequate for esophageal, gastric, and bowel obstructions, but patients with neurologic diseases, head or neck cancers, or lesions of the proximal esophagus should be managed with tube feeding [50]. For short periods of tube feeding, a nasogastric tube is sufficient, but for long-term tube feeding, longer than four weeks, endoscopic gastrostomy is the gold standard [51,52]. The decision to perform an endoscopic gastrostomy depends on the expected survival of each patient. Although several predictive models are available [53,54], the decision often relies on clinical judgment, demanding extensive experience in long-term enteral nutrition and multidisciplinary teams led by gastroenterologists. In the context of obesity, another facet of malnutrition, endoscopic therapies such as intragastric balloons and more recent procedures further illustrate the expanding role of endoscopy in nutritional management [55,56]. In the near future, endoscopic procedures for treating obesity and metabolic diseases will be as available as pharmacotherapy or surgery.

This Special Issue of *Nutrients* encompasses most aspects of the relationships between nutrition, the digestive system, and clinical gastroenterology. Altogether, these scientific, clinical, and technical dimensions demonstrate that gastroenterology is intrinsically tied to nutrition. The active involvement of gastroenterologists and digestive surgeons is essential to the practice of clinical nutrition and the comprehensive care of patients with digestive diseases.
